# Temperature‐driven color lightness and body size variation scale to local assemblages of European Odonata but are modified by propensity for dispersal

**DOI:** 10.1002/ece3.6596

**Published:** 2020-07-22

**Authors:** Daniel Acquah‐Lamptey, Martin Brändle, Roland Brandl, Stefan Pinkert

**Affiliations:** ^1^ Faculty of Biology Department of Ecology – Animal Ecology Philipps‐Universität Marburg Marburg Germany; ^2^ Ecology & Evolutionary Biology Yale University New Haven CT USA

**Keywords:** Bergmann's rule, dispersal, freshwater insects, habitat–stability–dispersal hypothesis, local assemblages, macrophysiology, Odonata, thermal adaptation, thermal melanism hypothesis

## Abstract

1. Previous macrophysiological studies suggested that temperature‐driven color lightness and body size variations strongly influence biogeographical patterns in ectotherms. However, these trait–environment relationships scale to local assemblages and the extent to which they can be modified by dispersal remains largely unexplored. We test whether the predictions of the thermal melanism hypothesis and the Bergmann's rule hold for local assemblages. We also assess whether these trait–environment relationships are more important for species adapted to less stable (lentic) habitats, due to their greater dispersal propensity compared to those adapted to stable (lotic) habitats.
2. We quantified the color lightness and body volume of 99 European dragon‐ and damselflies (Odonata) and combined these trait information with survey data for 518 local assemblages across Europe. Based on this continent‐wide yet spatially explicit dataset, we tested for effects temperature and precipitation on the color lightness and body volume of local assemblages and assessed differences in their relative importance and strength between lentic and lotic assemblages, while accounting for spatial and phylogenetic autocorrelation.
3. The color lightness of assemblages of odonates increased, and body size decreased with increasing temperature. Trait–environment relationships in the average and phylogenetic predicted component were equally important for assemblages of both habitat types but were stronger in lentic assemblages when accounting for phylogenetic autocorrelation.
4. Our results show that the mechanism underlying color lightness and body size variations scale to local assemblages, indicating their general importance. These mechanisms were of equal evolutionary significance for lentic and lotic species, but higher dispersal ability seems to enable lentic species to cope better with historical climatic changes. The documented differences between lentic and lotic assemblages also highlight the importance of integrating interactions of thermal adaptations with proxies of the dispersal ability of species into trait‐based models, for improving our understanding of climate‐driven biological responses.

## Graphical abstract

We quantified the color lightness and body volume of 99 European dragon‐ and damselflies (Odonata) and combined this trait information with survey data for 518 local assemblages across Europe to test whether trait‐environment relationships previously reported at coarse spatial scales also hold for spatially explicit local assemblages. We show that the color lightness of odonates assemblages increased and body size decrease with increasing temperature across Europe, supporting the thermal melanism hypothesis and Bergmann's rule, respectively. Accounting for differences in the dispersal/recolonization ability between lentic and lotic species and the phylogenetic relationships of species indicated that both groups show similar trait‐environment relationships in the phylogenetically predicted part of traits (evolutionary responses), but differ in their responses to (recent) historical climatic changes.

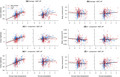

## 1 INTRODUCTION

Understanding the processes that shape species’ distributions and the composition of assemblages is central to ecological research (Brown & Maurer, 1987; Cavender‐Bares, Kozak, Fine, & Kembel, 2009; McGill, Enquist, Weiher, & Westoby, 2006; Ricklefs, 2004). A straightforward approach to gain a process‐based understanding is to investigate functional traits that link the physiology of a species with the ambient environment in which the species occurs (Chown, Gaston, & Robinson, 2004; Violle et al., 2007). Ectothermic species must absorb thermal energy from their environment to be active and to maintain fundamental physiological processes, including growth and reproduction (Angilletta, 2009; Huey & Kingsolver, 1989). Therefore, ectotherms have evolved several behavioral (e.g., wing‐whirring or basking; Corbet, 1980; May, 1979) and morphological adaptations to the climate in which they live (Angilletta, 2009; May, 1976).

Two of the most important morphological traits that influence the distribution of ectothermic organisms are probably their surface color, particularly color lightness (melanism), and body size. Fundamental physical principles link both traits to the heat gain and loss of an organism (Clusella‐Trullas, van Wyk, & Spotila, 2007; Shelomi, 2012). On the one hand, melanization of the cuticle determines the absorption of solar radiation and hence heat gain, a mechanism referred to as thermal melanism (Clusella‐Trullas et al., 2007; Gates, 1980; Kalmus, 1941). On the other hand, since an increase in body size implies a reduction of the surface area to volume ratio, larger bodies are able to retain heat more efficiently than smaller bodies (Shelomi, 2012). Besides thermoregulation, greater melanization increases resistance against pathogens, by enhancing the structural integrity of cells (Gloger's rule, Rapoport, 1969; Wilson, Cotter, Reeson, & Pell, 2001) and a larger body size is advantageous under dry conditions, as a lower surface area to volume ratio reduces water loss through the cuticle (Kühsel, Brückner, Schmelzle, Heethoff, & Blüthgen, 2017; Remmert, 1981).

While the color lightness and body size of a species should reflect the climate in which it can live (Willmer & Unwin, 1981), the extent to which that species realizes the potential environmental niche depends on its dispersal. Important differences in a species’ ability and propensity to disperse are related to the stability of their respective habitats (Southwood, 1977). In general, species restricted to spatially and temporally less stable habitats with higher dispersal abilities (Pellissier, 2015; Southwood, 1977) evolved set of adaptations (behavioral and morphological, Corbet, 1980) that facilitate persistence or the (re)colonization of habitats (Southwood, 1962), which are reflected in larger geographical ranges, stronger gene flow between populations and the ability to cope with climatic changes (Arribas et al., 2012; Hof, Brändle, & Brandl, 2006; Marten, Brändle, & Brandl, 2006; Pinkert et al., 2018). Freshwaters provide an ideal model system to test the predictions of this “habitat–stability–dispersal hypothesis” (Hof et al., 2012; Southwood, 1977). In the northern hemisphere, lentic water bodies (e.g., ditches and lakes) are ephemeral and date back to the Pleistocene, whereas the locations of rivers and streams (lotic waters) that carry water throughout the year have remained largely unaltered since the Mesozoic (Bohle, 1995 and sources therein). In contrast, though some lakes are very old (reviewed in Hutchinson, 1957), lotic habitats are on average more persistent over space and time than lentic habitats (Martens, 1997).

Recent studies have shown that the ecological differences between species adapted to lentic and lotic habitats carry a phylogenetic signal (Letsch, Gottsberger, & Ware, 2016). Moreover, these differences have found to be associated with contrasting biogeographical and diversification patterns between the two groups (Abellán, Millán, & Ribera, 2009; Hof, Brändle, & Brandl, 2008). For instance, Dehling, Hof, Brändle, and Brandl (2010) showed that the richness of lotic animals decreases from southern to northern Europe, whereas the richness of lentic animals is highest in central Europe. A broadly similar pattern has been reported for the richness of lentic and lotic Odonata (dragonflies and damselflies) on a global scale (Kalkman et al., 2008). Thus, in contrast to almost all other Odonata, the two youngest families (Coenagrionidae and Libellulidae; Rehn, 2003) that constitute the majority of lentic species globally (Kalkman et al., 2008) are disproportionally diverse in temperate climates. This suggests stronger trait–environment relationships in odonates of lentic than lotic habitats due to the greater ability of the former to cope with past climatic changes (Arribas et al., 2012; Grewe, Hof, Dehling, Brandl, & Brändle, 2013; Pinkert et al., 2018). However, despite strong support for an impact of species’ dispersal ability on biogeographical patterns, to what extent dispersal can modify trait–environment relationships remains largely unexplored.

Analyses of the large‐scale patterns of interspecific variation in physiological traits offer a powerful approach to elucidate the general processes that shape biodiversity patterns (Chown et al., 2004). These macrophysiological inferences based on the assumption that the explanations for large‐scale diversity patterns are found at lower levels of biological organization, as functional traits influence the fundamental physiological rates of individuals and populations whereas the consequences thereof play an important role in determining a species’ fundamental niche (Gaston & Blackburn, 2000). On the one hand, previous physiological studies on few species (Brakefield & Willmer, 1985; Harris, McQuillan, & Hughes, 2013) and local scale studies (e.g., along elevational gradients) have reported strong links between physiological trait and the environment, but these are often limited in spatial extent (Brehm, Zeuss, & Colwell, 2018; Dufour et al., 2018; Peters, Peisker, Steffan‐Dewenter, & Hoiss, 2016; Xing et al., 2018). On the other hand, most of the studies conducted so far on the interspecific variation of color lightness and body size in ectothermic species over large geographical ranges are based on expert range maps generated by interpolating species occurrence records across suitable habitats (e.g., Pinkert, Brandl, & Zeuss, 2017; Zeuss, Brandl, Brändle, Rahbek, & Brunzel, 2014; Zeuss, Brunzel, & Brandl, 2017; but see Bishop et al., 2016). Hence, previous evidence of color‐ and size‐based thermoregulation has three important limitations. First, although at geographical scales expert range maps are generally considered to allow robust estimations of the full environmental range of species, the underlying distribution information tends to overestimate species’ real distributional ranges (Hurlbert & Jetz, 2007; Merow, Wilson, & Jetz, 2017). Second, the inherent spatial structure of expert range maps has been shown to inadvertently generate spurious spatial patterns for the richness and mean trait values of assemblages (Hawkins et al., 2017). Third, distribution data with a coarse resolution generate “synthetic” assemblages of species that do not necessarily form local assemblages. For instance, expert range maps typically also include records of populations that may no longer exist (or never existed) and pool species from different habitat types. Therefore, whether the previously documented relationships of color lightness and body size with climate also scale to the local assemblage level remains largely unexplored.

In this study, we investigated trait–environment relationships using spatially explicit survey data for local assemblages of dragon‐ and damselflies (Odonata) across Europe. Specifically, according to the thermal melanism hypothesis and Bergmann's rule sensu* lato,* we expected (a) an increase in the color lightness of local assemblages of odonates with increasing temperature. If color lightness and body size are also involved in pathogen resistance and desiccation tolerance, we expect that (b) local assemblages of odonates are darker and smaller in more humid climates. In addition, given that adaptations to spatially and temporally less stable habitats allow lentic species to better cope with climatic changes (habitat–stability–dispersal hypothesis), we predicted that (c) the slopes of these relationships would be stronger for lentic than for lotic assemblages.

## 2 MATERIAL AND METHODS

### 2.1 Distribution data

**FIGURE 1:**
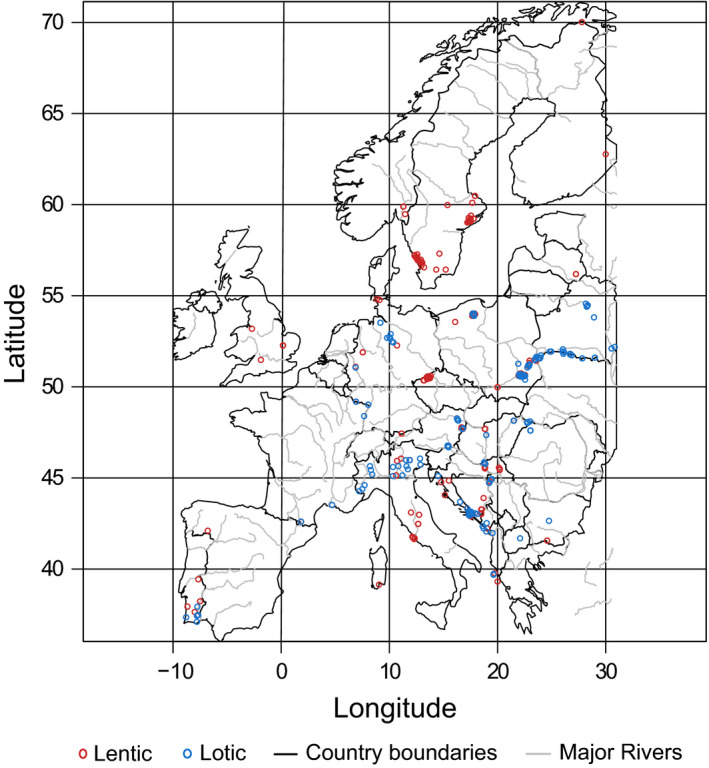
Distribution of (lentic = 337, lotic = 181) assemblages odonates across Europe. Lentic assemblages are indicated with red circles, lotic assemblages with blue circles, and the main European rivers in gray. The black outlines are country boundaries in the study region. The proportion of lotic habitats is higher in southern and central Europe. The space inside each circle represents the area for which the climate data were aggregated (a radius of ~1 km around the location of each community)

Information on water body location and type (i.e., lentic or lotic) and the composition of local assemblages of odonates across Europe were compiled from data obtained in an extensive literature survey (Appendix 1). Only records of breeding species were included, to obtain species sets associated with the considered water bodies. Breeding records covered tandem pairs, ovipositing females, larvae, exuviae, and recently emerged adults (Bried, Dillon, Hager, Patten, & Luttbeg, 2015), resulting in 5,703 records of 99 species of odonates and 524 local assemblages across 28 European countries. After assemblages with less than three species were excluded (to obtain reliable estimates of assemblage means), the final dataset comprised of 518 local assemblages of dragon‐ and damselflies (Figure 1; 337 lentic, 181 lotic).

### 2.2 Trait data

Following the most common approach used in the analysis of morphological traits based on digital images (Pinkert et al., 2017; Zeuss et al., 2014, 2017), we calculated the average color lightness and body volume of species using drawings of European Odonata (Dijkstra & Lewington, 2006). To prepare images for the analysis, the body (head, abdomen, and thorax) in scanned drawings of species’ dorsal body surfaces (24‐bits, sRGB, 1,200 dpi resolution) was cropped out and saved to separate files using functions of Adobe Photoshop CS2. Based on these images, the average color of the pixels of an image across the red, green, and blue channels was calculated as an estimate of the color lightness of a species (8‐bit gray values ranging from 0: absolute black to 255: pure white). In addition, these images were scaled with the magnification factor provided in Dijkstra and Lewington (2006) and used to calculate body volume in cm^3^ (*π* × [½ length of pixel row]^2^ × pixel edge length) as an estimate of the body size of a species based on the assumption that odonates generally have a cylindrical body form. The calculations were performed using functions of the R package *png* (Urbanek, 2013). Body volume instead of linear size measures, such as wing length, head width, and body length, was used because as a three‐dimensional measure it allows for a more realistic estimate of the body mass of a species (Kühsel et al., 2017). Note that previous studies showed that the color lightness and body volume estimates are correlated between drawings from different sources and between males and females (Pinkert et al., 2017; Zeuss et al., 2017). Subsequently, the average color lightness and body volume were calculated across the species of each local assemblage.

### 2.3 Environmental data

Based on the predictions of the thermal melanism hypothesis and Bergmann's rule, we considered annual mean temperature as a predictor of geographical patterns in the color lightness and body size of the assemblages of odonates. In addition, annual precipitation (AP) was included as a predictor because of the protective function of melanin against pathogens under humid climates (e.g., Delhey, 2019; Rapoport, 1969; Stelbrink et al., 2019) and the hypothesized advantage of lower water loss in large insects under dry climates (Kühsel et al., 2017; Remmert, 1981). We considered only annual mean temperature and annual precipitation, rather than all 19 commonly used bioclimatic variables, to facilitate interpretations of their effects and comparability with other studies (e.g., Pinkert et al., 2017; Zeuss et al., 2017). Nevertheless, the two variables contributed strongly to the overall trends in temperature and precipitation from a principal component analysis based on the correlation of 19 commonly used bioclimatic variables (Table S1). Climate variables used in the analysis were extracted from climate data with a resolution of 2.5 arcminutes (retrieved from www.chelsa‐climate.org; Karger et al., 2017, 2018), based on the geographical coordinates of the assemblages included in our analyses (with a buffer radius of approximately 1 km).

### 2.4 Statistical analyses

All statistical analyses and calculations were conducted in R (version 3.5.1, R Core Team, 2016).

Previous studies found that the color lightness and body size, as well as the habitat preference of European odonates, carry a phylogenetic signal (Letsch et al., 2016; Pinkert et al., 2017; Zeuss et al., 2014). Based on a recent phylogeny of the European odonates (Pinkert et al., 2018), we, therefore, partitioned the total variance of the color lightness and body volume into a phylogenetic component and a species‐specific component using Lynch's comparative method (Lynch, 1991), implemented in the R package *ape* (Paradis, Claude, & Strimmer, 2004). For data and methodology on the phylogeny of the European odonates, see Pinkert et al. (2018). The phylogenetic component represents the variation in color lightness and body volume predicted by the phylogenetic relationships of the species, whereas the species‐specific component is the difference of the observed trait estimate from the phylogenetically predicted part. The advantage of this method is that it allows assessing the effect of phylogenetic signals in traits (i.e., P‐component) that is often neglected as a source of bias, in addition to the model results that have been corrected for phylogenetic autocorrelation (i.e., S‐component).

Tests for trait–environment relationships were performed using single and multiple ordinary least‐squares regression models, with the average color lightness and body size of Odonata assemblages as dependent variables and climatic variables as independent variables. Differences in the slopes of the relationships of color lightness and body size with climatic variables between lentic and lotic habitats were determined by fitting interaction terms between the independent variables and habitat type. In all models, independent variables were scaled and centered (z‐standardized) to facilitate their comparison. We checked mulitcollinearity among predictors, using the *vif* function of the R package *car* (Table S2; Fox et al., 2016).

Since spatial autocorrelation in the survey data could violate the assumptions of our statistical models, that is, that all data points are independent of each other, spatial correlograms of the model residuals were calculated using functions of the R package *ncf* (Bjornstad, 2016). These correlograms indicated significant spatial autocorrelation in our data. Therefore, all analyses were repeated using spatial autoregressive error models (Dormann, 2007) that included a spatial distance weight according to the model‐specific point of spatial independence (extracted from spatial correlograms shown in Figures S1 and S2).

## 3 RESULTS

### 3.1 Trait–environment relationships

In all multiple regression models, the color lightness of the assemblages of odonates increased with increasing annual mean temperature and body volume decreased with increasing annual mean temperature (Table 1). In all multiple regression models, the color lightness of the assemblages of odonates was not affected by annual precipitation, but except for phylogenetically corrected models, body volume increased with increasing annual precipitation. These results were consistent with the results of single regression models (except that single regression of the average and phylogenetically predicted part of the variation in the color lightness and annual mean temperature were not significant) and with the results of models that accounted for spatial autocorrelation (Table 1). The two climate predictors together explained up to between 20% and 31% of the variation in color lightness and between 2% and 4% of the variation in body volume (Table 1, see Table S3 for regression models for individual habitat types).

**TABLE 1:** Effect sizes (*z*‐scores) and the explained variance of predictor variables from single and multiple regressions (*r* ^2^/*R* ^2^) of the average, phylogenetic, and species‐specific components of the average color lightness and body volume of 518 assemblages of European odonates with z‐standardized temperature and precipitation variables

Model		Trait	Predictor	Average		S‐component		P‐component	
				*Z*‐score	*r* ^2^/*R* ^2^	*Z*‐score	*r* ^2^/*R* ^2^	*Z*‐score	*r* ^2^/*R* ^2^
Ordinary least‐squares regression	Single	Color lightness	AMT	**14.28**	.28	**11.44**	.20	**11.88**	.22
			AP	**5.14**	.05	**3.41**	.02	**2.58**	.01
		Body volume	AMT	–1.59	.01	**–2.97**	.02	–1.33	.00
			AP	**2.25**	.01	−0.25	.00	**2.55**	.01
	Multiple	Color lightness	AMT	**13.12**	.29	**10.80**	.20	**11.55**	.22
			AP	1.68		0.33		–0.74	
		Body volume	AMT	**–2.40**	.02	**–3.04**	.02	**–2.21**	.02
			AP	**2.89**		0.68		**3.11**	
Spatial autoregressive error models	Single	Color lightness	AMT	**11.62**	.30	**9.87**	.21	**10.73**	.24
			AP	**2.69**	.14	1.47	.09	1.17	.08
		Body volume	AMT	–1.77	.03	**–2.50**	.02	–1.60	.03
			AP	**2.24**	.03	0.77	.01	**2.45**	.03
	Multiple	Color lightness	AMT	**11.32**	.31	**9.57**	.21	**10.64**	.24
			AP	1.71		0.50		−0.06	
		Body volume	AMT	–**2.15**	.04	–**2.59**	.02	**–2.00**	.04
			AP	**2.56**		1.04		**2.74**	

Note

In addition, regression models (Nagelkerke pseudo‐*r* ^2^/*R* ^2^) calculated with a spatial dependency weight are given. Significant relationships (*p* < .05) are shown in bold. The predictors are annual mean temperature (AMT) and annual precipitation (AP). The P‐component represents the phylogenetically predicted part of the respective trait, and S‐component represents the respective deviation of the average trait from the P‐component.

### 3.2 Differences in trait–environment relationships between habitat types

**FIGURE 2:**
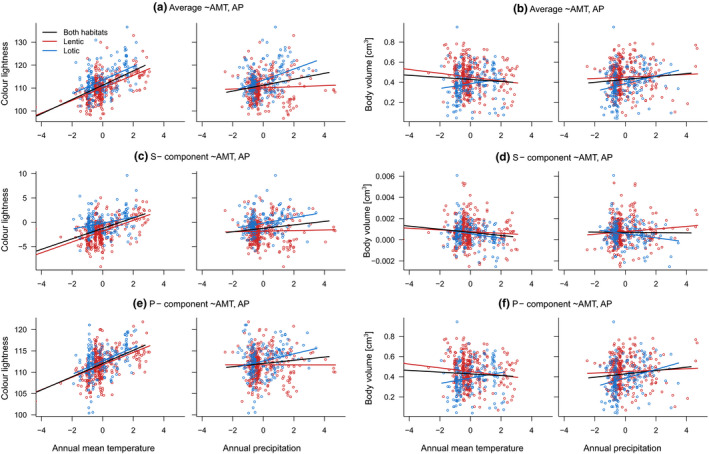
Scatterplots of the average (a, b), species‐specific (c, d), and phylogenetic (e, f) components of the average color lightness and body volume of (all habitats = 518, lentic = 337, lotic = 181) assemblages of European odonates and z‐standardized annual mean temperature, and annual precipitation. The color of the dots indicates the habitat type of the assemblages. Lines indicate regression lines of ordinary least‐squares models. The color lightness ranges from 0 (absolute black) to 255 (pure white). The P‐component represents the phylogenetically predicted part of the trait, and S‐component represents the respective deviation of the average trait from the P‐component

The relative importance of annual mean temperature and annual precipitation, as well as the slopes of the considered trait–environment relationships, differed between lentic and lotic assemblages (Figure 2). The two groups mostly responded similarly to annual mean temperature, and all the relationships that were only significant in lotic assemblages were responses to annual precipitation (see Table S4 for single regression models).

**TABLE 2:** Individual slopes and standard error of the predictor variables from multiple regressions (*R* ^2^) of the average, phylogenetic, and species‐specific components of the average color lightness and body volume of (lentic = 337, lotic = 181) assemblages of European odonates with *z*‐standardized environmental variables

Model	Trait	Component	Predictor	Slope ± *SE* for lentic	Slope ± *SE* for lotic	*R* ^2^
Ordinary least‐squares regression	Color lightness	Average	AMT	**3.0 × 10^0^ ± 2.8 × 10^–1^**	**2.7 × 10^0^ ± 4.4 × 10^–1^**	.29
			AP	1.4 × 10**^–^** ^1^ ± 2.9 × 10**^–^** ^1^	**8.6 × 10^–^** ^1^ ** ± 4.2 × 10^–1^**	
		S‐component	AMT	**1.2 × 10^0^ ± 1.2 × 10^–1^**	**6.6 × 10^–1^ ± 1.9 × 10^–1^**	.21
			AP	3.2 × 10^–2^ ± 1.3 × 10^–1^	2.6 × 10^–1^ ± 1.8 × 10^–1^	
		P‐component	AMT	**1.6 × 10^0^ ± 1.7 × 10^–1^**	**1.7 × 10^0^ ± 2.7 × 10^–1^**	.22
			AP	–9.7 × 10^–2^ ± 1.7 × 10^–1^	–1.5 × 10^–1^ ± 2.5 × 10^–1^	
	Body volume	Average	AMT	**–2.5 × 10^–2^ ± 8.3 × 10^–3^**	–1.2 × 10**^–^** ^2^ ± 1.3 × 10**^–^** ^2^	.03
			AP	6.3 × 10**^–^** ^3^ ± 8.5 × 10**^–^** ^3^	**3.6 × 10^–2^ ± 1.2 × 10^–2^**	
		S‐component	AMT	–9.9 × 10^–4^ ± 6.4 × 10^–5^	–1.7 × 10^–4^ ± 1.0 × 10^–4^	.03
			AP	**1.3 × 10^–4^ ± 6.5 × 10^–5^**	–9.3 × 10^–5^ ± 9.5 × 10^–5^	
		P‐component	AMT	**–2.3 × 10^–2^ ± 8.3 × 10^–3^**	–1.1 × 10^–2^ ± 1.3 × 10^–2^	.04
			AP	6.7 × 10^–3^ ± 8.5 × 10^–3^	**3.9 × 10^–2^ ± 1.3 × 10^–2^**	
Spatial autoregressive error models	Color lightness	Average	AMT	**2.9 × 10^0^ ± 3.1 × 10^–1^**	**2.6 × 10^0^ ± 4.6 × 10^–1^**	.31
			AP	3.0 × 10**^–^** ^1^ ± 3.1 × 10**^–^** ^1^	7.4 × 10^–1^ ± 4.3 × 10^–1^	
		S‐component	AMT	**1.2 × 10^0^ ± 1.3 × 10** ^–^ **^1^**	**6.2 × 10^–1^ ± 2.0 × 10^–1^**	.22
			AP	7.8 × 10^–2^ ± 1.3 × 10^–1^	2.3 × 10^–1^ ± 1.9 × 10^–1^	
		P‐component	AMT	**1.6 × 10^0^ ± 1.8 × 10** ^–^ **^1^**	**1.7 × 10^0^ ± 2.7 × 10^–1^**	.24
			AP	4.1 × 10^–2^ ± 1.9 × 10^–1^	–1.5 × 10^–1^ ± 2.6 × 10^–1^	
	Body volume	Average	AMT	**–2.2 × 10^–2^ ± 8.8 × 10^–3^**	–1.4 × 10**^–^** ^2^ ± 1.3 × 10^–2^	.05
			AP	8.1 × 10^–3^ ± 9.1 × 10^–3^	**3.3 × 10^–2^ ± 1.3 × 10^–2^**	
		S‐component	AMT	–7.8 × 10^–5^ ± 6.8 × 10^–5^	–1.4 × 10^–4^ ± 1.0 × 10^–4^	.04
			AP	**1.8 × 10^–4^ ± 7.0 × 10^–5^**	–9.1 × 10^–5^ ± 9.7 × 10^–5^	
		P‐component	AMT	**–2.2 × 10^–2^ ± 8.8 × 10^–3^**	–1.4 × 10^–2^ ± 1.3 × 10^–2^	.05
			AP	8.4 × 10^–3^ ± 9.2 × 10^–3^	**3.6 × 10^–2^ ± 1.3 × 10^–2^**	

Note

In addition, regression models (Nagelkerke pseudo‐*R* ^2^) calculated with a spatial dependency weight are given. Shaded cells indicate significant differences in the slopes of these regressions between lotic and lentic assemblages. Slopes that are significant from zero (*p* < .05) are shown in bold. The predictors are annual mean temperature (AMT) and annual precipitation (AP). The P‐component represents the phylogenetically predicted part of the trait, and S‐component represents the respective deviation of the average trait from the P‐component.

Except for one response of body size, the slopes of all relationships of climate variables with the average as well as the phylogenetically predicted part of the variation in the traits were similar in their strength between the two groups. By contrast, responses of the species‐specific part of the variation in color lightness and body size to climate were mostly stronger in lentic compared to lotic assemblages (Table 2). Similarly, the direction of the slopes of the relationships only differed between species‐specific part of the variation in body size and annual precipitation for both ordinary least‐squares regression and spatial autoregressive error models (Table 2).

## 4 DISCUSSION

Our results demonstrate that the spatial variation in color lightness and body size (i.e., volume) of assemblages of odonates across Europe is mainly driven by temperature. In line with the predictions of the thermal melanism hypothesis and Bergman's rule sensu* lato*, our results showed that the analyzed assemblages in warmer regions were consistently composed of, on average, lighter colored and smaller species of dragon‐ and damselflies compared to assemblages in cooler regions. Our continent‐wide yet spatially explicit assessment of these relationships reconciles previous macroecological (Pinkert et al., 2017; Zeuss et al., 2014, 2017) and experimental (e.g., May, 1991; Samejima & Tsubaki, 2010; reviewed in Clusella‐Trullas et al., 2007) evidence indicating the general importance of mechanistic links of color lightness and body size with the physiology and distribution of ectotherm species. In addition to the overall importance of color‐ and size‐based thermoregulation, our comparison of the trait–environment relationships of lentic and lotic assemblages of odonates revealed that the strength and relative importance of the climatic drivers of color lightness and body size vary considerably between species with high and low dispersal/recolonization ability.

Our study clearly showed that traits involved in thermoregulation influence the composition of dragon‐ and damselfly assemblages across Europe. According to the thermal melanism hypothesis, darker ectotherms are at an advantage in cool regions because of color‐based heat gain, and lighter ectotherms in warm regions because they reflect more solar radiation. In support of this hypothesis, we found that the color lightness of Odonata assemblages increased with increasing temperature. The results of our analyses based on survey data together with the similar geographical patterns in color lightness reported for assemblages of other ectothermic organisms at large geographical scales (Clusella‐Trullas, Terblanche, Blackburn, & Chown, 2008; Heidrich et al., 2018; Schweiger & Beierkuhnlein, 2015; Stelbrink et al., 2019; Xing et al., 2018; Zeuss et al., 2014), confirm that thermal melanism is a mechanism of major importance in ectothermic organisms across regions and scales. Furthermore, consistent with the predictions of Bergmann's rule sensu* lato*, we found that the average body size of assemblages of odonates decreased with increasing temperature. Even though a recent macroecological study by Zeuss et al. (2017) found support for Bergmann's rule in European odonates, its support in insects is generally equivocal (Shelomi, 2012), especially in studies conducted at small spatial and taxonomic scales. These contradictions in the results obtained at different scales have recently motivated debate about the reliability of large‐scale assemblage‐level studies, as it has been demonstrated that the type of distribution information on which most macroecological studies are based can purely by chance result in geographical patterns of species’ traits (Hawkins et al., 2017). Despite temperature explained a comparatively low variance in body size (c.f. Zeuss et al., 2017), our findings support Bergmann's rule sensu* lato* in European odonates. Our support for both the thermal melanism hypothesis and Bergmann's rule using spatially explicit survey data for European odonates shows that the findings of studies based on expert range maps are robust to pseudoreplications of co‐occurrences and the inherent geographical structures of species distributions (Hawkins et al., 2017).

Moreover, we also documented clear differences between species adapted to lentic and lotic habitats regarding the strength of the slopes of the considered trait–environment relationships and the relative importance of climatic drivers. Contrary to our third prediction, most of the relationships of average color lightness and body size with temperature were equally strong between lentic and lotic assemblages. However, decomposing variations in color lightness and body size showed that this is the result of similar responses of the phylogenetically predicted part of the traits of lentic and lotic species to climate, whereas relationships of the species‐specific part of the traits were mostly stronger in lentic assemblages. Several studies have suggested that lentic species are stronger dispersers (e.g., Grewe et al., 2013; Hof et al., 2006; Marten et al., 2006) due to the negative relationship between habitat persistence and dispersal propensity (Southwood, 1962). Species adapted to lentic habitats are assumed to be closer to an equilibrium with ambient temperature (Dehling et al., 2010; Pinkert et al., 2018) and hence should dominate in recently recolonized regions (e.g., formerly glaciated northern parts of Europe; Pinkert et al., 2018). Accordingly, color‐ and size‐based thermoregulation together with high dispersal ability may have been hypothesized to cause contrasting biogeographical patterns between species adapted to lentic and lotic habitats over historical and evolutionary time scales (Hof et al., 2008; Pinkert et al., 2018). In fact, the distributional success and high diversity of lentic species in temperate regions seem to result not only from higher dispersal/recolonization ability but also from an adaptive color and body size evolution by lentic lineages. Our results suggest that adaptive color and body size are of similar importance for lentic and lotic species over evolutionary time scales, but that historical responses modified trait–environment relationships, with lentic species responding stronger to recent climatic changes than lotic species.

In light of previous zoogeographical and phylogeographical studies on dragon‐ and damselflies (Abellán et al., 2009; Kalkman et al., 2008; Pinkert et al., 2018; Sternberg, 1998), the documented differences in the trait–environment relationships of lentic and lotic species suggest that thermal melanism favors the colonization of lineages of odonates in temperate climates. It has long been hypothesized that odonates are of tropical evolutionary origin and that only a few lineages acquired the ability to colonize and persist in temperate regions (e.g., Tillyard, 1917 p. 295). In a recent study, we found that the phylogenetic diversity of European Odonata assemblages decreased from the southwest to the northeast of the continent and that this pattern was mainly driven by the contemporary temperature (Pinkert et al., 2018). Latitudinal gradients of decreasing family or genus richness have been shown for odonates at the global scale (a simple proxy for the diversity of lineages; Kalkman et al., 2008). Furthermore, recent studies have documented a strong phylogenetic signal in the color lightness of odonates and butterfly assemblages as well as differences in the importance of thermal melanism between butterfly families and associated these differences with a lower importance color‐based thermoregulation in tropical lineages (Pinkert et al., 2017; Stelbrink et al., 2019; Zeuss et al., 2014). Therefore, our finding that phylogenetically predicted part of the variation in color lightness and body size is strongly driven by temperature suggested that color‐ and size‐based thermoregulation might have played a central role in the adaptation to colder climates, whereas most Odonata lineages retained their initial tropical niche (see also Pinkert & Zeuss, 2018). Besides the differences in the strengths of the relationships of color lightness and body size with temperature, our results show that the relative importance of temperature versus precipitation in shaping the geographical patterns of these traits differs between lentic and lotic assemblages. Although both annual mean temperature and annual precipitation consistently drove overall geographical patterns in the color lightness and body volume of assemblages of odonates, lotic, but not lentic species seem to have an additional advantage of a higher size‐based desiccation tolerance (Entling, Schmidt‐Entling, Bacher, Brandl, & Nentwig, 2010) that also constrain their ability to thermoregulate via this trait. Specifically, we found that lotic assemblages in regions of lower precipitation were on average smaller than those in humid regions, which points to body size as an adaptation to water loss through the body surface (Kühsel et al., 2017). Furthermore, we showed that species adapted to lotic habitats were significantly larger in regions that are both warm and wet. This finding supports the predictions of Gloger's rule (Wilson et al., 2001), which have been generally strongly supported by several large‐scale studies (Pinkert et al., 2017; Stelbrink et al., 2019; Zeuss et al., 2014). Although studies have shown that melanization impacts desiccation resistance (Parkash, Rajpurohit, & Ramniwas, 2008; Parkash, Sharma, & Kalra, 2009), we are cautious about interpreting a potential color‐based protection against water loss for two reasons; first, the environmental gradient of the study sites did not include extreme humid or dry regions, and second, in our study annual precipitation was not an important driver of the variations in color lightness European Odonata assemblages.

## 5 CONCLUSION

Our study highlights the importance of the mechanistic links of color lightness and body size with the temperature regime which shapes the biogeographical patterns of dragon‐ and damselflies (Odonata). Color‐ and size‐based thermoregulation was by far the dominant mechanisms shaping the composition of assemblages of odonates, although other functions of body size and color lightness seemed to influence the geographical patterns of both traits to some extent. The consistency of our findings together with the results of a number of macroecological analyses underlines the general importance of thermal melanism and Bergmann's rule for ectothermic organisms. However, besides highlighting the crucial role of traits involved in thermoregulation in shaping the distribution of odonate species, our results indicate that difference in species’ dispersal propensities embedded in the spatio‐temporal stability of their habitats contributes to explaining the scatter around the considered trait–environment relationships as well as to differences in the relative contributions of climatic predictors. Thus, thermal adaptations seem to be of similar evolutionary importance for lentic and lotic species but a greater dispersal ability of the former in combination with the climatic history of Europe seems to have allowed them better cope with historical climatic changes.

## CONFLICT OF INTEREST

The authors declare no conflict of interest.

## AUTHOR CONTRIBUTION

**Daniel Acquah‐Lamptey:** Conceptualization (lead); Data curation (lead); Formal analysis (lead); Methodology (lead); Writing‐original draft (lead); Writing‐review & editing (equal). **Martin Brändle:** Conceptualization (supporting); Supervision (supporting); Writing‐review & editing (equal). **Roland Brandl:** Conceptualization (equal); Methodology (equal); Supervision (lead); Writing‐review & editing (equal). **Stefan Pinkert:** Conceptualization (lead); Data curation (lead); Formal analysis (lead); Methodology (lead); Supervision (supporting); Writing‐review & editing (equal).

## Supporting information

Supplementary Material

## Data Availability

Data for this study are archived with Dryad Data Repository at https://doi.org/10.5061/dryad.k98sf7m43.
