# Supplementary material for: Temperature‐driven color lightness and body size variation scale to local assemblages of European Odonata but are modified by propensity for dispersal
Source: Ecol Evol. 2020 Jul 22;10(16):8936–48. doi: 10.1002/ece3.6596 (PMC7452777; doi:10.1002/ece3.6596)
Supplement: Supplementary file 1 — Supplementary Material [file ECE3-10-8936-s001.docx]

**— Supporting information —**

**Temperature-driven colour lightness and body size variation scale to local assemblages of European Odonata but are modified by propensity for dispersal**

Daniel Acquah-Lamptey^1*^, Martin Brändle^1^, Roland Brandl^1^**,** Stefan Pinkert^1, 2^

**AFFILIATIONS**

^1^Faculty of Biology, Department of Ecology - Animal Ecology, Philipps-Universität Marburg, Karl-von-Frisch-Straße 8, 35043 Marburg, Germany.

^2^Ecology & Evolutionary Biology, Yale University, 06511 CT, New Haven, USA.

^*^Correspondence: Daniel Acquah-Lamptey, Faculty of Biology, Department of Ecology - Animal Ecology, Philipps-Universität Marburg, Karl-von-Frisch-Straße 8, 35043 Marburg, Germany. E-mail: [dacquahlamptey@gmail.com](mailto:dacquahlamptey@gmail.com)

**TABLE S1** Variable contributions of principal component analysis based on the correlation of 19 commonly used temperature and precipitation variables calculated using the *prcomp* function of the R-package *stats*. Bioclimatic variables used in the analysis were extracted from climate data with a resolution of 2.5 arcminutes (retrieved from www.chelsa-climate.org; Karger et al., 2017; Karger et al., 2018), based on the geographical coordinates of the assemblages of European odonates (with a buffer radius of ~1 km). Variable contributions were calculated by singular value decomposition of the (centred and scaled) data matrix.

|  | Temperature | | Precipitation | |
| --- | --- | --- | --- | --- |
| Bioclimatic variables | PC 1 | PC 2 | PC 1 | PC 2 |
| Annual mean temperature | 11.62 | 9.45 |  |  |
| Mean diurnal temperature | 9.07 | 11.79 |  |  |
| Isothermality | 0.01 | 1.81 |  |  |
| Temperature seasonality | 8.83 | 10.64 |  |  |
| Max temperature of warmest month | 1.93 | 32.68 |  |  |
| Min temperature of coldest month | 15.09 | 0.28 |  |  |
| Temperature annual range | 11.69 | 8.02 |  |  |
| Mean temperature of wettest quarter | 5.77 | 8.19 |  |  |
| Mean temperature of driest quarter | 12.81 | 0.03 |  |  |
| Mean temperature of warmest quarter | 8.48 | 15.29 |  |  |
| Mean temperature of coldest quarter | 14.71 | 1.81 |  |  |
| Annual precipitation |  |  | 24.57 | 0.82 |
| Precipitation of wettest month |  |  | 22.08 | 2.9 |
| Precipitation of driest month |  |  | 3.32 | 26.13 |
| Precipitation seasonality |  |  | 0.16 | 25.54 |
| Precipitation of wettest quarter |  |  | 22.22 | 2.89 |
| Precipitation of driest quarter |  |  | 7.49 | 20.79 |
| Precipitation of warmest quarter |  |  | 0.25 | 19.07 |
| Precipitation of coldest quarter |  |  | 19.89 | 1.83 |
| Eigenvalues | 6.34 | 2.59 | 3.83 | 3.28 |
| Standard deviation | 2.52 | 1.61 | 1.96 | 1.81 |
| proportion of variance | 57.64 | 23.53 | 47.86 | 41.06 |
| Cumulative proportion of variance | 57.64 | 81.17 | 47.86 | 88.92 |

**TABLE S2** Variance inflation factors of predictor variables in regression models of average colour lightness and body volume (Tables 1) of assemblages of European odonates with z-standardized temperature and precipitation variables.

| Predictors | Both | Lentic | Lotic | Interaction |
| --- | --- | --- | --- | --- |
| Annual mean temperature | 1.10 | 1.00 | 1.58 | 1.13 |
| Annual precipitation | 1.10 | 1.00 | 1.58 | 1.13 |

**TABLE S3** Effect sizes (z-scores) and the explained variance of predictor variables from single and multiple regressions (*r*^2^/*R*^2^) of the average, species-specific and phylogenetic components of the average colour lightness and body volume of 337 lentic and 181 lotic assemblages of European odonates with z-standardized temperature and precipitation variables. In addition, regression models (Nagelkerke pseudo-*r*^2^/*R*^2^) calculated with a spatial dependency weight are given. Significant relationships (*p* < 0.05) are shown in bold. The predictors are: annual mean temperature (AMT), and annual precipitation (AP). The P - component represents the phylogenetically predicted part of the respective trait and S - component represents the respective deviation of the average trait from the P - component.

|  |  |  | | Average | | | | S - component | | | | P - component | | | |
| --- | --- | --- | --- | --- | --- | --- | --- | --- | --- | --- | --- | --- | --- | --- | --- |
|  |  |  | Lentic |  | Lotic |  | Lentic |  | Lotic |  | Lentic |  | Lotic |  |  |
| Model | Trait | | Predictor | Z-score | *r*^2^/*R*^2^ | Z-score | *r*^2^/*R*^2^ | Z-score | *r*^2^/*R*^2^ | Z-score | *r*^2^/*R*^2^ | Z-score | *r*^2^/*R*^2^ | Z-score | *r*^2^/*R*^2^ |
| Ordinary least-squares regression | Single | Colour lightness | AMT | **10.97** | 0.26 | **7.44** | 0.24 | **10.01** | 0.23 | **3.91** | 0.08 | **9.49** | 0.21 | **6.45** | 0.19 |
|  |  |  | AP | 0.85 | 0.00 | **5.73** | 0.20 | 0.49 | 0.00 | **3.54** | 0.07 | –0.02 | 0.00 | **3.22** | 0.06 |
|  |  | Body volume | AMT | –**2.51** | 0.02 | **2.12** | 0.03 | –1.26 | 0.00 | –**2.8** | 0.04 | –**2.45** | 0.02 | **2.36** | 0.03 |
|  |  |  | AP | 0.88 | 0.00 | **3.53** | 0.06 | 1.94 | 0.01 | –**2.53** | 0.03 | 0.93 | 0.00 | **3.85** | 0.08 |
|  | Multiple | Colour lightness | AMT | **9.5** | 0.21 | **5.45** | 0.19 | **9.98** | 0.23 | **2.22** | 0.09 | **9.5** | 0.21 | **5.45** | 0.19 |
|  |  |  | AP | –0.64 |  | –0.54 |  | –0.09 |  | 1.51 |  | –0.64 |  | –0.54 |  |
|  |  | Body volume | AMT | –**2.52** | 0.02 | 0.11 | 0.08 | –1.39 | 0.02 | –1.58 | 0.05 | –**2.52** | 0.02 | 0.11 | 0.08 |
|  |  |  | AP | 1.1 |  | **2.99** |  | 2.03 |  | –1.07 |  | 1.1 |  | **2.99** |  |
| Spatial autoregressive error | Single | Colour lightness | AMT | **10.25** | 0.28 | **4.56** | 0.28 | **10.04** | 0.23 | **2.46** | 0.12 | **9.2** | 0.26 | **6.39** | 0.19 |
|  |  |  | AP | 0.57 | 0.06 | **2.73** | 0.23 | –0.05 | 0.05 | 1.92 | 0.11 | 0.13 | 0.07 | 1.28 | 0.08 |
|  |  | Body volume | AMT | –**2.5** | 0.03 | 1.29 | 0.03 | –1.07 | 0.01 | –**2.92** | 0.04 | –**2.45** | 0.03 | 1.3 | 0.04 |
|  |  |  | AP | 0.97 | 0.02 | **3.15** | 0.07 | **2.68** | 0.03 | –**2.48** | 0.03 | 1.02 | 0.02 | **3.3** | 0.08 |
|  | Multiple | Colour lightness | AMT | **10.21** | 0.28 | **3.71** | 0.28 | **10.04** | 0.23 | 1.83 | 0.12 | **9.22** | 0.26 | **5.4** | 0.19 |
|  |  |  | AP | 1.04 |  | 1.26 |  | –0.10 |  | 1.04 |  | 0.68 |  | –0.56 |  |
|  |  | Body volume | AMT | –**2.48** | 0.04 | –0.03 | 0.07 | –0.96 | 0.03 | –1.61 | 0.05 | –**2.43** | 0.04 | –0.02 | 0.08 |
|  |  |  | AP | 0.93 |  | **2.65** |  | **2.61** |  | –1.07 |  | 0.96 |  | **2.81** |  |

**TABLE S4** Individual slopes and standard error of predictor variables from single regression (*r*^2^) of the average, species-specific and phylogenetic components of the average colour lightness and body volume of 337 lentic and 181 lotic assemblages of European odonates with z-standardised environmental variables. In addition, regression models (Nagelkerke pseudo-*r*^2^) calculated with a spatial dependency weight are given. Shaded cells indicate significant differences in the slopes of these regressions between lotic and lentic assemblages. Slopes that are significant from zero (*p* < 0.05) are shown in bold. The predictors are: annual mean temperature (AMT); and annual precipitation (AP). The P - component represents the phylogenetically predicted part of the trait and S - component represents the respective deviation of the average trait from the P - component.

| Model | Trait | Component | Predictor | Slope ± SE for lentic | Slope **±** SE for lotic | *r*^2^ |
| --- | --- | --- | --- | --- | --- | --- |
| Ordinary least-square regression | Colour lightness | Average | AMT | **3.0 × 10^0^ ± 2.8 × 10^–1^** | **3.3 × 10^0^ ± 3.5 × 10^–1^** | 0.28 |
|  |  | S - component | AMT | **1.2 × 10^0^ ± 1.2 × 10^–1^** | **8.2 × 10^–1^ ± 1.5 × 10^–1^** | 0.21 |
|  |  | P - component | AMT | **1.5 × 10^0^ ± 1.7 × 10^–1^** | **1.6 × 10^0^ ± 2.1 × 10^–1^** | 0.22 |
|  |  | Average | AP | 3.8 × 10^–1^ ± 3.3 × 10^–1^ | **2.5 × 10^0^ ± 3.8 × 10^–1^** | 0.08 |
|  |  | S - component | AP | 1.3 × 10^–1^ ± 1.4 × 10^–1^ | **6.6 × 10^–1^ ± 1.6 × 10^–1^** | 0.03 |
|  |  | P - component | AP | 2.6 × 10^–2^ ± 1.9 × 10^–1^ | **8.5 × 10^–1^ ± 2.2 × 10^–1^** | 0.03 |
|  | Body volume | Average | AMT | **–2.4 × 10^–2^ ± 8.3 × 10^–3^** | 1.1 × 10^–2^ ± 1.0 × 10^–2^ | 0.02 |
|  |  | S - component | AMT | –9.1 × 10^–5^ ± 6.4 × 10^–5^ | **–2.3 × 10^–4^ ± 7.9 × 10^–5^** | 0.02 |
|  |  | P - component | AMT | **–2.4 × 10^–2^ ± 8.4 × 10^–3^** | 1.4 × 10^–2^ ± 1.0 × 10^–2^ | 0.02 |
|  |  | Average | AP | 4.4 × 10^–3^ ± 8.5 × 10^–3^ | **2.8 × 10^–2^ ± 9.9 × 10^–3^** | 0.02 |
|  |  | S - component | AP | 1.3 × 10^–4^ ± 6.5 × 10^–5^ | **2.0 × 10^–4^ ± 7.5 × 10^–5^** | 0.02 |
|  |  | P - component | AP | 4.9 × 10**^–3^** ± 8.6 × 10^–3^ | **3.2 × 10^0^ ± 9.9 × 10^–3^** | 0.02 |
| Spatial autoregressive error models | Colour lightness | Average | AMT | **2.1 × 10^1^ ± 2.1 × 10^0^** | **2.5 × 10^1^ ± 2.0 × 10^0^** | 0.33 |
|  |  | S - component | AMT | **6.9 × 10^0^ ± 8.6 × 10^–1^** | **8.9 × 10^0^ ± 8.1 × 10^–1^** | 0.25 |
|  |  | P - component | AMT | **1.3 × 10^1^ ± 1.3 × 10^0^** | **1.3 × 10^1^ ± 1.2 × 10^0^** | 0.24 |
|  |  | Average | AP | **2.1 × 10^0^ ± 1.5 × 10^0^** | **4.9 × 10^0^ ± 1.7 × 10^0^** | 0.15 |
|  |  | S - component | AP | 9.4 × 10^–1^ ± 6.1 × 10^–1^ | 4.6 × 10^–1^ ± 7.2 × 10^–1^ | 0.09 |
|  |  | P - component | AP | 2.0 × 10^–1^ ± 8.6 × 10^–1^ | 1.8 × 10^0^ ± 1.0 × 10^0^ | 0.08 |
|  | Body volume | Average | AMT | –3.5 × 10^–2^ ± 6.1 × 10^–2^ | **–1.4 × 10^–1^ ± 5.9 × 10^–2^** | 0.05 |
|  |  | S - component | AMT | **–8.9 × 10^–4^ ± 4.5 × 10^–4^** | **–1.2 × 10^–3^ ± 4.3 × 10^–4^** | 0.02 |
|  |  | P - component | AMT | –2.9 × 10^–2^ ± 6.2 × 10^–2^ | **–1.3× 10^–1^ ± 6.0 × 10^–2^** | 0.02 |
|  |  | Average | AP | 5.6 × 10^–3^ ± 3.7 × 10^–2^ | **1.6 × 10^–1^ ± 4.4 × 10^–2^** | 0.05 |
|  |  | S - component | AP | **6.8 × 10^–4^ ± 2.9 × 10^–4^** | –4.9 × 10^–4^ ± 3.4 × 10^–4^ | 0.03 |
|  |  | P - component | AP | 8.1 × 10^–3^ ± 3.7 × 10^–2^ | **1.8 × 10^–1^ ± 4.4 × 10^–2^** | 0.05 |

**
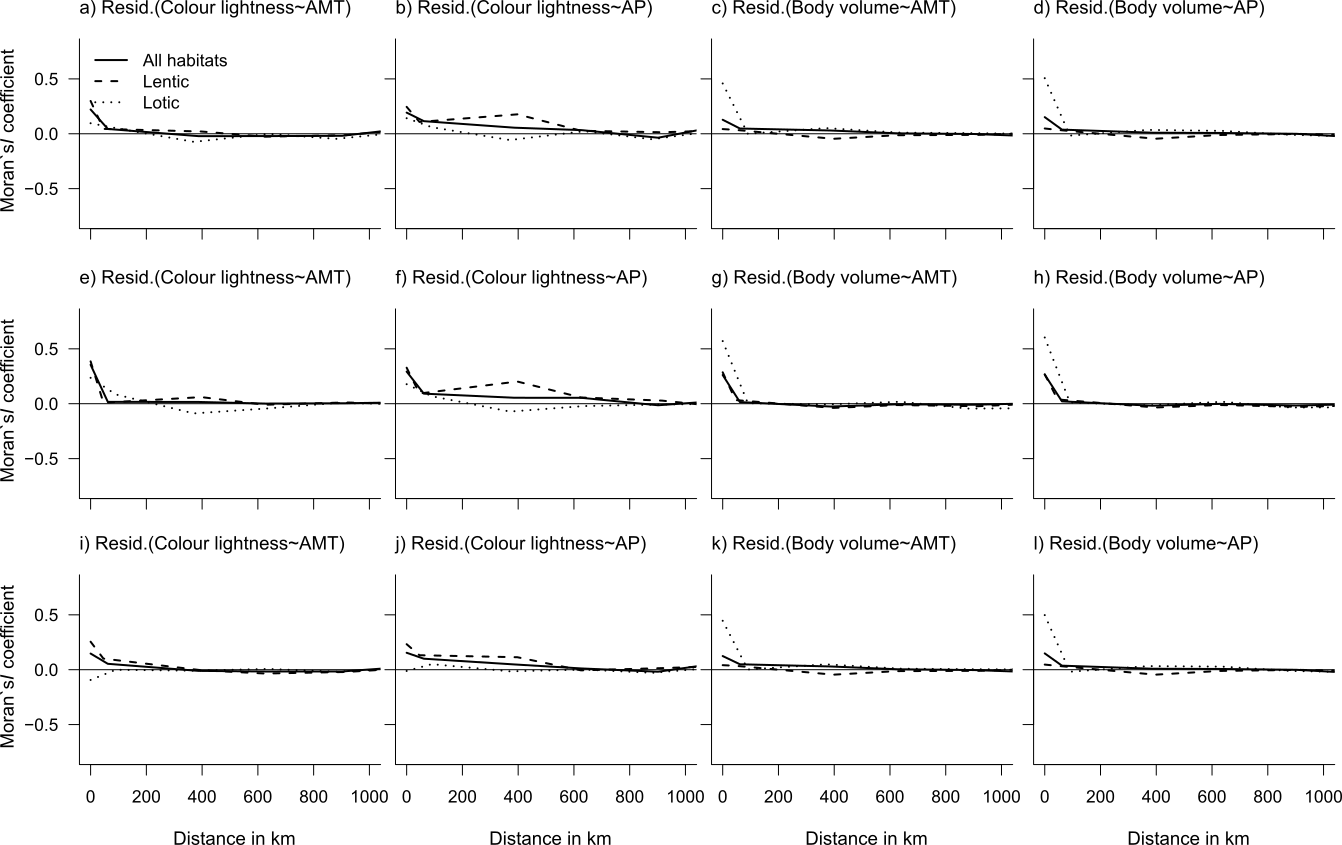
**

**FIGURE S1** Spatial correlogram of Moran's *I* coefficient of the residuals from the single regression model of the average, species-specific and phylogenetic components of the average colour lightness and body volume of assemblages of European odonates (all habitats = 518, lentic = 337, lotic = 181) with z-standardized temperature and precipitation variables. The abscissa is distance classes of assemblages and the ordinate is Moran's *I* coefficients where a value of zero indicates no autocorrelation. Moran's *I* coefficient of the residuals from regression models of the average (a – d), species-specific (e – h) and phylogenetic (i – l) components of colour lightness and body volume with annual mean temperature and annual precipitation respectively.

**
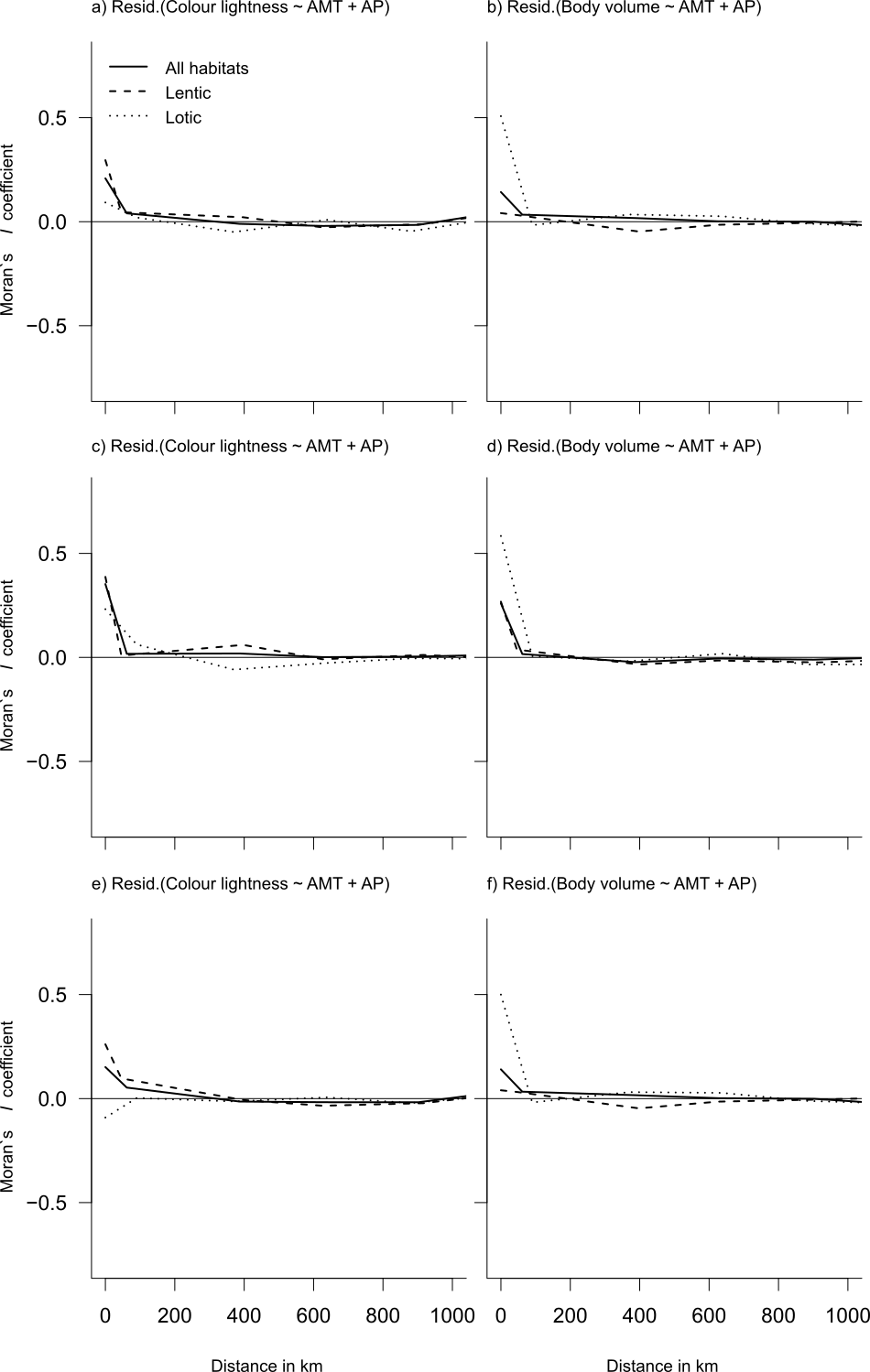
**

**FIGURE S2** Spatial correlogram of Moran's *I* coefficient of the residuals from the multiple regression model of the average, species-specific and phylogenetic components of the average colour lightness and body volume of assemblages of European odonates (all habitats = 518, lentic = 337, lotic = 181) with z-standardized temperature and precipitation variables. The abscissa is distance classes of assemblages and the ordinate Moran's *I* coefficients where a value of zero indicates no autocorrelation. Moran's *I* coefficient of the residuals from regression models of the average (a, b), species-specific (c, d) and the phylogenetic (e, f) component of colour lightness and body volume with annual mean temperature and annual precipitation.

**REFERENCES**

Karger, D. N., Conrad, O., Böhner, J., Kawohl, T., Kreft, H., Soria-Auza, R. W., … Kessler, M. (2017). Climatologies at high resolution for the earth’s land surface areas. *Scientific Data*, 4, 170122. https://doi.org/10.1038/sdata.2017.122

Karger, D. N., Conrad, O., Böhner, J., Kawohl, T., Kreft, H., Soria-Auza, R. W., … Kessler, M. (2018). Data from: Climatologies at high resolution for the earth’s land surface areas - Dryad Digital Repository. Retrieved July 9, 2018, https://doi:10.5061/dryad.kd1d4
